# Developmental effects on sleep–wake patterns in infants receiving a cow’s milk-based infant formula with an added prebiotic blend: a Randomized Controlled Trial

**DOI:** 10.1038/s41390-020-1044-x

**Published:** 2020-07-02

**Authors:** John Colombo, Susan E. Carlson, Cecilia Algarín, Sussanne Reyes, Maciej Chichlowski, Cheryl L. Harris, Jennifer L. Wampler, Patricio Peirano, Carol Lynn Berseth

**Affiliations:** 1 Schiefelbusch Institute for Life Span Studies and Department of Psychology, University of Kansas, Lawrence, KS USA; 2 Department of Dietetics and Nutrition, University of Kansas Medical Center, Kansas City, KS USA; 3 Sleep and Functional Neurobiology Laboratory, Institute of Nutrition and Food Technology (INTA), University of Chile, Santiago, Chile; 4 Nutrition Science, Department of Medical Affairs, Mead Johnson Nutrition, 2400 West Lloyd Expy, Evansville, IN 47721 USA; 5 Clinical Research, Department of Medical Affairs, Mead Johnson Nutrition, 2400 West Lloyd Expy, Evansville, IN 47721 USA

## Abstract

**Background:**

Few studies have evaluated nutritive effects of prebiotics on infant behavior state, physiology, or metabolic status.

**Methods:**

In this double-blind randomized study, infants (*n* = 161) received cow’s milk-based infant formula (Control) or similar formula with an added prebiotic blend (polydextrose and galactooligosaccharides [PDX/GOS]) from 14–35 to 112 days of age. Infant wake behavior (crying/fussing, awake/content) and 24-h sleep–wake actograms were analyzed (Baseline, Days 70 and 112). Salivary cortisol was immunoassayed (Days 70 and 112). In a subset, exploratory stool 16S ribosomal RNA-sequencing was analyzed (Baseline, Day 112).

**Results:**

One hundred and thirty-one infants completed the study. Average duration of crying/fussing episodes was similar at Baseline, significantly shorter for PDX/GOS vs. Control at Day 70, and the trajectory continued at Day 112. Latency to first and second nap was significantly longer for PDX/GOS vs. Control at Day 112. Cortisol awakening response was demonstrated at Days 70 and 112. Significant stool microbiome beta-diversity and individual taxa abundance differences were observed in the PDX/GOS group.

**Conclusions:**

Results indicate faster consolidation of daytime waking state in infants receiving prebiotics and support home-based actigraphy to assess early sleep–wake patterns. A prebiotic effect on wake organization is consistent with influence on the gut–brain axis and warrants further investigation.

**Impact:**

- Few studies have evaluated nutritive effects of prebiotics on infant behavior state, cortisol awakening response, sleep–wake entrainment, and gut microbiome.
- Faster consolidation of daytime waking state was demonstrated in infants receiving a prebiotic blend in infant formula through ~4 months of age.
- Shorter episodes of crying were demonstrated at ~2 months of age (time point corresponding to age/developmental range associated with peak crying) in infants receiving formula with added prebiotics.
- Results support home-based actigraphy as a suitable method to assess early sleep–wake patterns.
- Prebiotic effect on wake organization is consistent with influence on the gut–brain axis and warrants further investigation.

## Introduction

Infant state regulation, reflecting responsivity to everyday environmental stimuli, matures and consolidates with age. A predominant example is crying, considered a universal form of early communication, which promotes parental/caregiver proximity and enhances chances of survival.^1^ The daily time spent crying and fussing in infants typically increases from birth, peaks ~6–8 weeks of age, and subsequently decreases with age.^2^ Other important characteristics of behavioral development include sleep pattern consolidation and response to stress, both maturing rapidly during infancy. Infant sleep–wake patterns undergo progressive nocturnal sleep consolidation and maturation of wake rhythm during the first 6 months of age. Whereas newborn infant sleeps 16–17 h/day, by 6 months the amount drops to 13–14 h, with the longest sleep period extending to 6 h.^3^ Sufficient non-rapid eye movement (non-REM) sleep,^4^ along with proper nutrition,^5^ can impact brain development. Changes in percentages of waking and sleeping states, each also corresponding to a specific brain functional state, occur with advancing age; for example, active sleep (the precursor of REM sleep) decreases, quiet sleep (non-REM sleep) increases, and indeterminate sleep decreases throughout the first 6 months and disappears in a short time.^6^

Cortisol, a hormone involved in regulation of circadian-driven activities,^7^ may also be linked to sleep quality and stress management. Relation of cortisol secretion to sleep duration, feeding time, and maternal cortisol at birth is still controversial;^8^ however, a diurnal pattern in parallel with day/night rhythm develops from 3 to 6 months of age.^9^ The cortisol awakening response (CAR) is characterized as: cortisol levels reach a nadir by late evening (typically at waking/sleep transition and sleep onset); begin to increase by midnight; and finally peak ~30 min after awakening. CAR emerges from 2 to 6 months of age and can be assessed using salivary cortisol measures.^10,11^

Bi-directional communication linking the brain and gut function, known as the gut–brain axis, has prompted interest in examining the impact of nutrition and gut microbiome composition on behavior, brain development, learning, and cognition. Prebiotics are dietary components that have been defined as: “a substrate that is selectively utilized by host microorganisms conferring a health benefit.”^12^ In healthy term infants, we have previously demonstrated a prebiotic blend of polydextrose (PDX) and galactooligosaccharides (GOS) (4 g/L, 1:1 ratio) in infant formula was well tolerated, supported normal growth, promoted softer stools closer to that of breastfed infants, and had a bifidogenic effect closer to breast milk when compared to infants fed a formula without the added prebiotic blend.^13–17^ This same blend of PDX and GOS has also been associated with development of typical behavioral states in preterm infants who demonstrated a significant reduction in excessive crying.^18^ These data are consistent with the hypothesis that prebiotics may have an impact on broader brain-mediated behaviors.

Preclinical data using a rodent sleep model has recently demonstrated effects of dietary prebiotics on sleep physiology. Beneficial REM sleep rebounded more quickly and reductions in disruption of the diurnal rhythm of core body temperature and dysbiosis (using three measures of alpha diversity) were demonstrated in a feeding group randomized to receive the dietary blend of PDX and GOS.^19^ Data from the same study further linked sleep physiology and gut metabolites, including secondary bile acids (e.g., hyodeoxycholic acid) and known neuroactive gut metabolites.^20^ Overall, the study diet that had the blend of PDX and GOS was demonstrated to modulate stress and sleep in the preclinical model. To date, no studies have evaluated the nutritive effects, safety, and tolerance of prebiotics on typical infant behavioral regulation. Consequently, the objective of this study was to evaluate sleep measures and infant state in infants randomized to receive cow’s milk-based infant formula with or without an added prebiotic blend of PDX and GOS (4 g/L, 1:1 ratio) through ~4 months of age.

## Methods

### Study design and participants

In this multicenter, double-blind, randomized, controlled, parallel-group, prospective trial (ClinicalTrials.gov: https://clinicaltrials.gov/ct2/show/NCT02118935), healthy infants (14-35 days of age) were recruited at nine clinical sites in the United States. This study was designed to assess behavior using a parental diary to measure infant state, home-based actigraphy to assess sleep–wake patterns, and CAR. In a limited subset of participants, the association between early-life microbiome and the PDX and GOS blend was also examined. Participants were enrolled between July 2014 and November 2015. Parents or guardians provided written informed consent prior to enrollment. The research protocol and informed consent forms observing the Declaration of Helsinki (including October 1996 amendment) were approved by The University of Kansas Medical Center–Human Research Protection Program (Kansas City, KS, USA) and Schulman IRB (now known as Advarra, Columbia, MD). The study complied with good clinical practices.

Eligible infants were singleton births at 37–42 weeks gestational age with birth weight ≥2500 g and had a history of normal growth. Mothers had chosen to primarily feed infant formula prior to study enrollment. Eligible infants received ≥75% of the recommended caloric intake from marketed infant formula over the 24 h prior to randomization to study formula. Exclusion criteria included maternal history of prenatal illicit drug use or clinically significant psychiatric disease; infant history of underlying metabolic or chronic disease or congenital malformation likely to interfere with the ability to ingest food, normal growth, and development, or participant evaluation; feeding difficulties or history of formula intolerance; evidence of or concerns for growth difficulties; immunodeficiency; and planned use of probiotics during the study period.

### Randomization and study group allocation

The study sponsor created a computer-generated, sex-stratified randomization schedule provided in sealed consecutively numbered envelopes for each study site. Study formula was assigned by opening the next sequential envelope from the appropriate set at the study site. Study formulas, each designated by two unique codes known only to the sponsor, were dispensed to parents at each study visit prior to completion or withdrawal. Neither the product labels nor the sealed envelopes allowed direct unblinding by the study site. Personnel responsible for monitoring the study were also blinded to study product identification. Blinding for a participant could be broken by study sponsor personnel in the event of a medical emergency in which knowledge of the study formula was critical to the participant’s management. In this study, it was not necessary to break the study code prematurely. Participants were randomly assigned to receive one of two study formulas (Mead Johnson Nutrition, Evansville, IN): (1) a routine cowʼs milk-based infant formula that had a prebiotic blend of PDX (Litesse® Two Polydextrose; Danisco) and GOS (Vivinal® GOS; Friesland Foods Domo) (4 g/L, 1:1 ratio; marketed Enfamil®; PDX/GOS) or (2) a similar formula that had no added prebiotic blend (Control; previously marketed Enfamil) from study randomization up to 112 days of age. Study formulas were provided as ready-to-use liquids (20 cal/fluid ounce) and had docosahexaenoic acid at 17 mg/100 kcal and arachidonic acid at 34 mg/100 kcal.

### Study outcomes

Study visits corresponded to Baseline (14–35 days of age), randomization to study formula (4–8 days following Baseline), Day 70 (±7 days), and Day 112 (±7 days). At the randomization visit, inclusion/exclusion criteria were verified. Baseline diary and actigraphy data were received, and participants were assigned to a study formula group. The primary outcome in this pilot study was assessment of the nutritive effects of a prebiotic-containing infant formula on infant behavioral state and sleep–wake patterns using a validated parent-reported diary and sleep actigraphy at Baseline, Day 70, and Day 112 as the primary variables to assess early behavioral indicators of tolerance. If an infant was sick at the time of a scheduled assessment, the evaluation was postponed until he/she recovered. Secondary outcomes included achieved anthropometrics (body weight, length, and head circumference) and a 24-h parent recall of stool characteristics (frequency/day and consistency scaled from 1 to 5: hard, formed, soft, unformed or seedy, watery), gassiness, and fussiness recorded at all study time points. A 24-h recall of formula intake (fluid oz/day) was collected at Days 70 and 112. CAR was also reported at Days 70 and 112. Adverse events were collected throughout the study period and coded according to specific event (e.g., otitis media, colic, etc.) and the body system involved.

#### Infant behavior diary

Parents/caregivers reported “crying/fussing” and “awake and content” behavior using a validated paper diary^21–23^ over a consecutive 72-h period to evaluate infant state. Diary recordings were completed at Baseline (recording began the day after that study visit), Day 70 (between 60 and 77 days of age), and Day 112 (between 102 and 119 days of age). Each 24-h period was represented by four 6-h time bars (5-min subdivisions) corresponding to: morning (6:00 a.m. to noon), afternoon (noon to 6:00 p.m.), evening (6:00 p.m. to midnight), and night (midnight to 6:00 a.m.) (see Supplemental Figure). Crying/fussing and awake/content behaviors were recorded in 5-min increments with parents encouraged to fill in the diary every 2–3 h, or at the same time as a repetitive activity, such as feeding or changing a diaper. Behaviors recorded as “other” (e.g., sleeping, eating, cannot recall) were not analyzed.

#### Actigraphy

Infants wore waterproof actigraph devices (Actiwatch 2, Philips Respironics, Bend, OR, USA) without removal (on leg or ankle) for a minimum of 3 consecutive days to continuously record periods of movement and rest. Actigraphy recordings occurred at Baseline (recording began the evening of that study visit), Day 70 (between 59 and 77 days of age), and Day 112 (between 101 and 119 days of age). Motor activity was not collected when a participant was sick/ill. Each 24-h interval was divided into nocturnal and diurnal periods. The nocturnal period began with the onset of the first sleep episode after 20:00 hours, which was followed for at least 30 consecutive minutes of sustained sleep; this period ended with the transition to the diurnal period, which started when the first wake episode appeared after 06:00 hours and was followed for at least 30 consecutive minutes of wakefulness. Actigraphic data was digitalized, stored for each successive 1-min interval, and processed on a min-by-min basis to produce individual actograms for each participant (Actiware, Philips Respironics). Typically the manufacturer’s software automatically determines the start of a sleep or wake epoch using a pre-set algorithm. Actigraphy has demonstrated good sensitivity (ability to detect sleep), but poor-to-fair specificity (ability to detect wake after sleep onset).^24^ Therefore, to avoid inaccurate detection of short sleep and wake episodes, often the primary source of errors using actigraphic recordings at different ages,^25^ we reassessed this first detection of sleep–wake episodes for each individual actogram as described previously.^26^ Briefly, shorter changes within sleep or wake episodes lasting at least 8 min were incorporated in the ongoing episode to generate a new sequence. For example, the sequence SSSSSSSSWWSWSSSS (S = sleep, W = wake) based on 1-min length would become SSSSSSSSSSSSSS. Sleep–wake variables for the whole 24-h period were total wake and sleep times. For nocturnal and diurnal periods, these variables include: wake-up and sleep onset times, total duration of time spent awake, mean duration and number of wake episodes, total duration of time spent asleep, mean duration and number of sleep episodes, latency to and duration of first nap, latency to second nap, latency to first wake episode, longest sleep episode duration during the first and second half of the night, and onset time of the longest sleep episode during the second half of the night.

#### Salivary cortisol

Participant saliva samples were collected at home within 48-h prior to Day 70 and 112 study visits directly upon first natural wakening and 30 min post wakening. Pooled saliva in the participant’s cheek or under the tongue (~1 mL/sample) was collected using a validated infant swab and tube system (SalivaBio Infant’s Swab, Salimetrics, Carlsbad, CA). Parents were instructed that the infant should have no feedings until after the second sampling. Sample tubes were refrigerated within 30 min of collection, kept frozen at or below −20 °C (within 4 h of collection), and salivary cortisol (μg/dL) was analyzed by enzyme immunoassay as described previously (Salimetrics, State College, PA).

#### Stool collection and microbiome analysis

In a subset of participants, a stool sample was received at the randomization visit and at Day 112. DNA isolation, enrichment, and sequencing were performed (Second Genome, South San Francisco, CA), PCR products were sequenced (Illumina MiSeq), and QIIME operational taxonomic units (OTUs) were assigned as previously described.^27^

### Statistical analysis

A sample size of 50 completed participants per feeding group was estimated to allow detection of an effect size of 0.57 (alpha = 0.05, two-tailed test, 80% power). Twenty-four-hour sleep–wake variables were averaged from collected motor activity data and analyzed by repeated-measures analysis of variance (ANOVA).

Infant state variables (durations and frequencies averaged within time points); achieved weight, length, and head circumference; and formula intake were analyzed by mixed models for repeated measures. Salivary cortisol was analyzed using paired *t* tests. Stool frequency was analyzed by ANOVA. Stool consistency was analyzed using the Cochran–Mantel–Haenszel test. Fussiness and gas were analyzed using the *χ*^2^ test. Study discontinuations and the incidence of adverse events were analyzed using Fisher’s exact test. For exploratory microbiome analysis, alpha-diversity (OTU richness and Shannon) and beta-diversity (Bray–Curtis) measures were analyzed by study group and time point. Taxa associated with study groups were determined.

## Results

### Participants

**Fig. 1:**
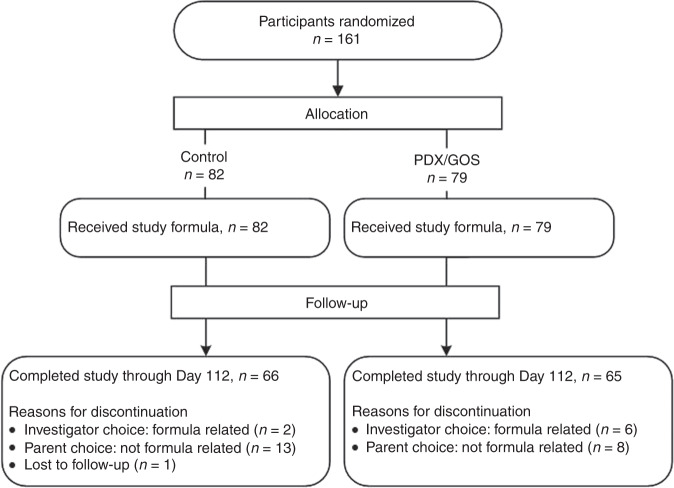
Study allocation. Flow of study participants.

A total of 161 participants were enrolled and randomized (Control: *n* = 82; PDX/GOS: *n* = 79) (Fig. 1). At study enrollment, anthropometric measures, age, and gender distribution were similar among groups (Table 1). No statistically significant group differences were detected for study discontinuation (Control: *n* = 16, 20%; PDX/GOS: *n* = 14, 18%) or discontinuation related to study formula (Control: *n* = 2, 2%; PDX/GOS: *n* = 6, 8%). Of the eight participants with discontinuation related to study formula, a study investigator elected to discontinue one participant and seven (4% of the total study population) were discontinued by a study investigator due to formula intolerance. The most common symptoms of formula intolerance were gas (Control: *n* = 1; PDX/GOS: *n* = 4), fussiness (PDX/GOS, *n* = 4), and emesis (PDX/GOS, *n* = 4). A total of 131 infants completed the study (Control: *n* = 66; PDX/GOS: *n* = 65).

**Table 1:** Infant characteristics at study entry.

	Study group	
	Control	PDX/GOS
Total number of participants	82	79
Number of males/females	43/39	42/37
Males^a^		
Weight (g)	4192 ± 81.1	4171 ± 73.3
Length (cm)	53.7 ± 0.3	53.6 ± 0.3
Head circumference (cm)	37.3 ± 0.2	37.4 ± 0.2
Females^a^		
Weight (g)	3963 ± 73.1	3712 ± 59.7
Length (cm)	52.9 ± 0.3	52.1 ± 0.3
Head circumference (cm)	36.7 ± 0.2	36.1 ± 0.2
Age at enrollment	23.4 ± 0.8	21.9 ± 0.8

^a^Mean ± standard error (SE).

### Infant state

**Fig. 2:**
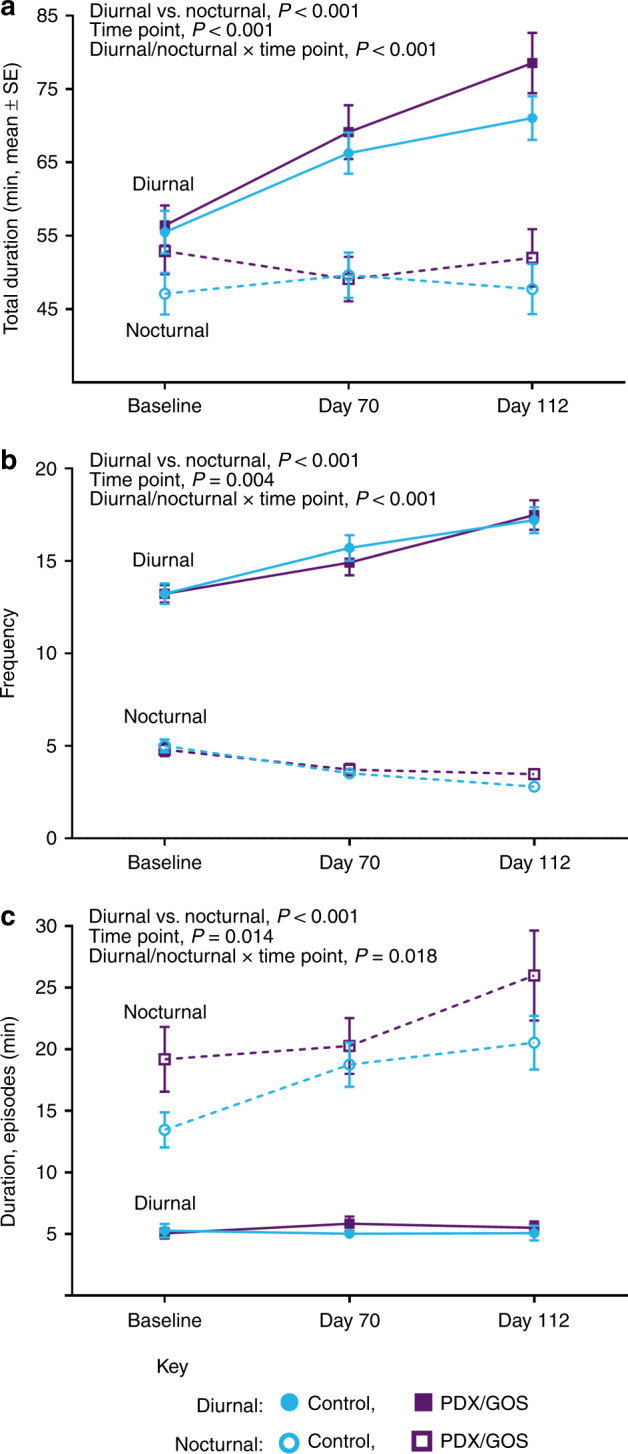
Awake and content behavior (mean ± SE). **a** Total duration (min), **b** frequency (number of episodes), and **c** average duration of episodes (min) as measured by parent-reported Barr diary. The respective sample sizes for the diurnal period at Baseline, Day 70, and Day 112 were: Control: 82, 69, 66 and PDX/GOS: 77, 66, 63. The respective sample sizes for the nocturnal period at Baseline, Day 70, and Day 112 were: Control: 77, 63, 53 and PDX/GOS: 70, 58, 49. Diurnal: Control, solid blue circles; PDX/GOS, solid purple squares and Nocturnal: Control, open blue circles; PDX/GOS, open purple squares.

Waking behavior characterized as “awake and content” is shown in Fig. 2. Total diurnal or total nocturnal duration (Fig. 2a) was ~50 min for each group at Baseline, but diverged over time for both groups (*P* < 0.001). Total nocturnal duration remained steady through Day 112, whereas total diurnal duration significantly increased through Days 70 and 112 (both group means were ~70–80 min). The frequency (Fig. 2b) of awake and content episodes was significantly higher during the diurnal vs. nocturnal period (*P* < 0.001). Nocturnal frequency decreased from Baseline to Day 112 (group means decreased from ~5 to 3), whereas diurnal frequency increased from Baseline to Day 112 (group means increased from ~13 to 17). Average duration of episodes (Fig. 2c) was significantly longer for nocturnal vs. diurnal at all study time points (*P* < 0.001). Whereas average diurnal episodes remained unchanged (group means ~5 min), average nocturnal episodes significantly increased from Baseline to Day 112 (*P* = 0.018).

**Fig. 3:**
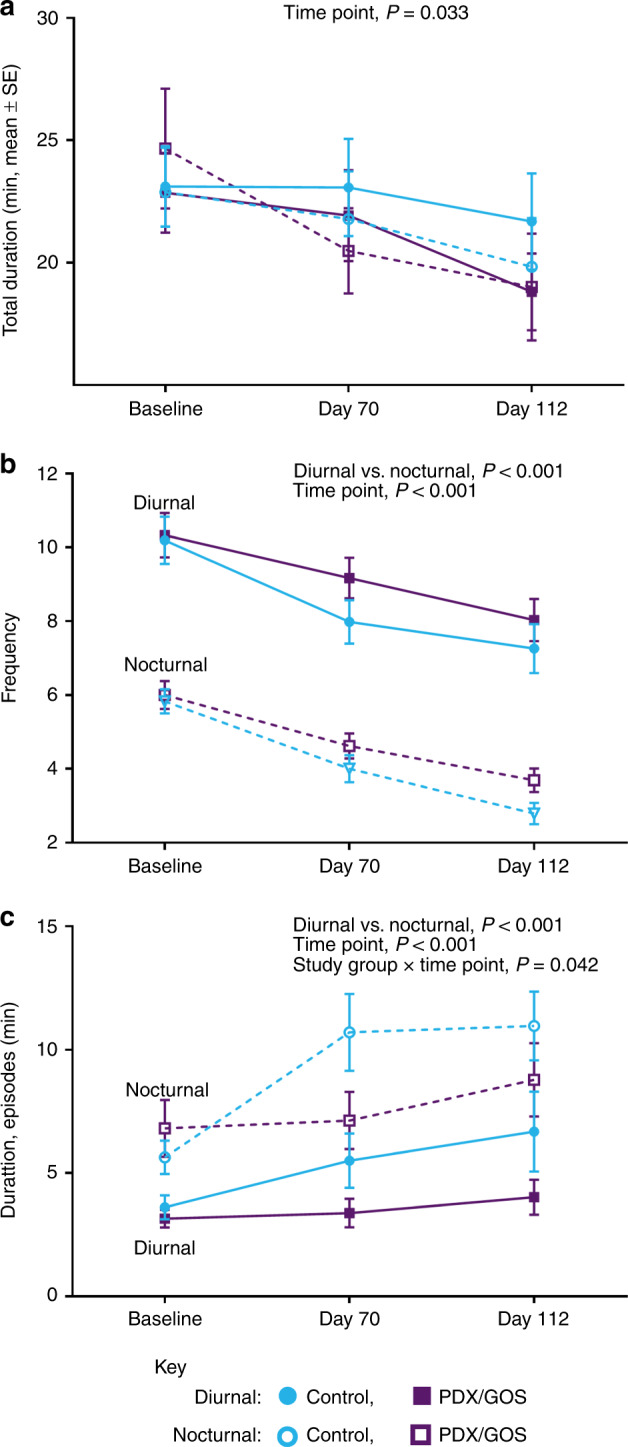
Crying and fussing behavior (mean ± SE). **a** Total duration (min), **b** frequency (number of episodes), and **c** average duration of episodes (min) as measured by parent-reported Barr diary. The respective sample sizes for the diurnal period at Baseline, Day 70, and Day 112 are Control: 78, 63, 61 and PDX/GOS: 75, 65, 61. The respective sample sizes for the nocturnal period at Baseline, Day 70, and Day 112 are Control: 77, 58, 47 and PDX/GOS: 70, 60, 52. Diurnal: Control, solid blue circles; PDX/GOS, solid purple squares and Nocturnal: Control, open blue circles; PDX/GOS, open purple squares.

Waking behavior characterized as “crying/fussing” is shown in Fig. 3. Total duration of crying/fussing (Fig. 3a) significantly decreased from Baseline to Day 112 (*P* = 0.033). Frequency of episodes (Fig. 3b) also significantly decreased by study end (*P* < 0.001). Frequency of nocturnal compared to diurnal episodes was lower from Baseline to Day 112 (*P* < 0.001). From Baseline to Day 112, average duration of episodes (Fig. 3c) was significantly longer for nocturnal vs. diurnal (*P* < 0.001) and increased with age (*P* < 0.001). A significant study group × time point interaction (*P* = 0.042) was explored by further analysis at each study time point: average duration of crying/fussing episodes were similar at Baseline, significantly shorter for the PDX/GOS compared to Control at Day 70 (*P* = 0.017) and continued the same trajectory at Day 112 (*P* = 0.089).

### Sleep–wake patterns

Diurnal, nocturnal, and 24-h sleep–wake variables by study time point and by group (mean ± SE) are presented in Table 2. For diurnal napping, the number and duration of nap episodes was similar between groups. The duration of 1st nap was also similar between groups; however, latency to 1st nap began to diverge at Day 70 and was significantly longer in the PDX/GOS vs. Control group by Day 112 (2.7 ± 0.1 vs. 2.2 ± 0.1; *P* = 0.023). Subsequent latency to 2nd nap since wake onset was significantly longer in the PDX/GOS vs. Control group at Day 112 (*P* = 0.028). For nocturnal waking, latency to 1st wake episode was significantly longer in the PDX/GOS vs. Control group at Baseline (*P* = 0.034) and Day 70 (*P* = 0.014), although the number and duration of episodes was similar between groups. A significant difference in total nocturnal sleep time was detected at Day 112 (Control: 7.3 ± 0.1, PDX/GOS 6.8 ± 0.1; *P* = 0.033), although no group differences were detected in mean duration of sleep episodes or duration of the longest sleep episode during the 1st half of the night. During the 2nd half of the night, the longest sleep episode duration was significantly longer at Day 70 (1.6 ± 0.1 vs. 1.3 ± 0.1; *P* = 0.034) and the onset time (h:min ± min) of the episode was significantly earlier by Day 112 (4:00 ± 7 vs. 4:23 ± 7; *P* = 0.014) in the PDX/GOS vs. Control group. For other common sleep–wake variables, with the exception of total wake time at Day 70 (Control: 14.2 ± 0.2, PDX/GOS 13.6 ± 0.2; *P* = 0.027), no group differences were detected at any study time point for: 24-h total wake or sleep time, total diurnal wake time; or total diurnal nap time.

**Table 2:** Diurnal, nocturnal, and 24-h sleep–wake variables by study time point and by group (mean ± SE).

	Baseline		Day 70		Day 112	
Sleep–wake variables^a^	Control	PDX/GOS	Control	PDX/GOS	Control	PDX/GOS
Diurnal (day) period						
Wake-up time (h:min ± min)	7:32 ± 8	7:26 ± 8	6:41 ± 7	6:47 ± 7	6:43 ± 6	6:36 ± 6
Total wake time (h)	10.2 ± 0.3	9.8 ± 0.3	12.0 ± 0.2	11.4 ± 0.2	11.6 ± 0.1	11.7 ± 0.1
Napping						
Number of naps	5.4 ± 0.2	5.1 ± 0.2	5.3 ± 0.2	5.0 ± 0.2	4.7 ± 0.2	4.5 ± 0.2
Duration of nap episodes (h)	0.8 ± 0.04	0.9 ± 0.04	0.6 ± 0.03	0.7 ± 0.03	0.7 ± 0.03	0.7 ± 0.03
Latency to 1st nap (h)	1.9 ± 0.1	1.8 ± 0.1	1.8 ± 0.1	2.2 ± 0.1	2.2 ± 0.1	2.7 ± 0.1^b^
Duration of 1st nap (h)	0.9 ± 0.1	1.1 ± 0.1	0.7 ± 0.1	0.8 ± 0.1	0.6 ± 0.1	0.8 ± 0.1
Latency to 2nd nap, since wake onset (h)	4.2 ± 0.2	4.1 ± 0.2	4.2 ± 0.2	4.3 ± 0.2	4.5 ± 0.2	5.2 ± 0.2^b^
Latency to 2nd nap, since the end of 1st nap (h)	1.3 ± 0.1	1.1 ± 0.1	1.5 ± 0.1	1.5 ± 0.1	1.5 ± 0.1	1.8 ± 0.1
Total nap (sleep) time (h)	4.2 ± 0.2	4.4 ± 0.2	3.2 ± 0.2	3.3 ± 0.2	3.0 ± 0.1	3.0 ± 0.1
Nocturnal (night) period						
Sleep onset time (h:min ± min)	22:05 ± 9	21:50 ± 9	21:50 ± 9	21:47 ± 9	21:14 ± 8	21:22 ± 8
Total sleep time (h)	7.1 ± 0.2	7.2 ± 0.2	6.4 ± 0.2	6.7 ± 0.2	7.3 ± 0.1	6.8 ± 0.1^b^
Duration of sleep episodes (h)	1.8 ± 0.1	1.9 ± 0.1	1.6 ± 0.1	1.8 ± 0.1	1.8 ± 0.1	1.6 ± 0.1
Longest sleep episode duration, 1st half of night (h)	2.7 ± 0.1	3.0 ± 0.1	3.2 ± 0.2	3.4 ± 0.2	3.7 ± 0.2	3.4 ± 0.2
Longest sleep episode duration, 2nd half of night (h)	2.1 ± 0.1	2.2 ± 0.1	1.3 ± 0.1	1.6 ± 0.1^b^	1.3 ± 0.1	1.3 ± 0.1
Onset time, longest sleep episode, 2nd half of night (h:min ± min)	4:17 ± 6	4:33 ± 7	4:14 ± 7	4:20 ± 8	4:23 ± 7	4:00 ± 7^b^
Waking						
Number of wake episodes	3.3 ± 0.1	3.0 ± 0.1	3.6 ± 0.2	3.3 ± 0.2	3.7 ± 0.2	3.8 ± 0.2
Duration of wake episodes (h)	0.8 ± 0.04	0.8 ± 0.04	0.6 ± 0.03	0.7 ± 0.03	0.6 ± 0.02	0.6 ± 0.02
Latency to first wake episode (h)	1.8 ± 0.1	2.2 ± 0.1^b^	2.0 ± 0.1	2.6 ± 0.1^b^	2.5 ± 0.2	2.4 ± 0.2
24-h period						
Total wake time (h)	12.4 ± 0.3	12.3 ± 0.3	14.2 ± 0.2	13.6 ± 0.2^b^	13.7 ± 0.2	14.0 ± 0.2
Total sleep time (h)	11.2 ± 0.2	11.7 ± 0.2	9.6 ± 0.2	9.9 ± 0.2	10.2 ± 0.2	9.9 ± 0.2

^a^Complete actigraphy data (all study time points) was available for *n* = 124 participants.

^b^Significant group difference at study time point, *P* < 0.05.

### Cortisol awakening response

The presence of CAR was demonstrated at Days 70 and 112 (Table 3) and was unrelated to sex, study time point, or study feeding group. Salivary cortisol (μg/dL, mean ± SE) significantly increased from first awakening to 30 min post wakening for both study groups at Days 70 and 112 (*P* ≤ 0.001).

**Table 3:** Salivary cortisol (μg/dL) at wakening and post wakening.

	Salivary cortisol (μg/dL)^a^		
Group (n)	Awakening	Post wakening	*P* value
Day 70			
Control (65)	0.599 ± 0.11	1.169 ± 0.18	<0.001
PDX/GOS (62)	0.563 ± 0.81	1.021 ± 0.15	<0.001
Day 112			
Control (58)	0.602 ± 0.09	0.869 ± 0.10	<0.011
PDX/GOS (57)	0.539 ± 0.10	0.887 ± 0.08	<0.001
Overall	0.576 ± 0.05	0.995 ± 0.07	<0.001

^**a**^Mean ± standard error (SE).

### Growth and tolerance

No significant group differences were observed for mean achieved weight, length, or head circumference throughout the study (data not shown). At enrollment, parent-reported gassiness and fussiness (data not shown) and stool characteristics were similar between groups (Table 4). No significant group differences in gassiness, fussiness, or study formula intake were detected at Days 70 and 112. No significant differences in mean (±SE) stool frequency (number/day) or stool consistency was detected in the Control vs. PDX/GOS group at any measured study time point. In stool consistency categories, soft or unformed or seedy were most commonly reported for participants in both study groups.

**Table 4:** Stool characteristics at Baseline, Day 70, and Day 112.

			Stool consistency, *n* (%)					
Group (*n*)	Stool frequency^a^	*P* value	Hard	Formed	Soft	Unformed or seedy	Watery	*P* value
Baseline								
Control (82)	3.0 ± 0.2	0.440	0 (0%)	2 (2%)	42 (51%)	36 (44%)	2 (2%)	0.937
PDX/GOS (79)	2.7 ± 0.2		0 (0%)	4 (5%)	38 (48%)	34 (43%)	3 (4%)	
Day 70								
Control (66)	2.1 ± 0.2	0.958	0 (0%)	1 (2%)	36 (57%)	24 (38%)	2 (3%)	0.395
PDX/GOS (65)	2.1 ± 0.2		0 (0%)	0 (0%)	41 (67%)	19 (31%)	1 (2%)	
Day 112								
Control (62)	1.9 ± 0.2	0.099	0 (0%)	0 (0%)	42 (70%)	16 (27%)	2 (3%)	0.915
PDX/GOS (60)	2.3 ± 0.2		0 (0%)	4 (7%)	30 (55%)	19 (35%)	2 (4%)	

^a^Mean ± standard error (SE) stool frequency. Means for Baseline, Day 70, and Day 112 are based on 24-h recall at study visits.

The number of participants for whom at least one medically confirmed adverse event was reported was significantly different between groups (Control: *n* = 45, 55%; PDX/GOS: *n* = 58, 73%; *P* = 0.021). The incidence of adverse events categorized within Cardiovascular; Eyes, Ear, Nose, and Throat; Metabolic and Nutrition; Musculoskeletal; Nervous System; Respiratory; Skin; or Urogenital systems were generally low with no statistically significant group differences for specific events. Within body as a whole, the overall incidence was significantly lower in the Control (*n* = 5, 6%) vs. the PDX/GOS group (13, 16%; *P* = 0.046); however, there were no significant differences between specific types of adverse events within this category. Within the gastrointestinal (GI) system the overall incidence was also significantly lower in the Control (*n* = 16, 20%) vs. the PDX/GOS group (29, 37%; *P* = 0.022). The most commonly reported specific adverse events were gastroesophageal (GE) reflux, gas, and constipation. There were no group differences in the incidence of GE reflux or gas; however, the incidence of constipation was significantly lower in the Control (*n* = 1, 1%) versus the PDX/GOS group (7, 9%; *P* = 0.032). Also within the GI system category, the incidence of diarrhea was low but significantly different between groups (Control: 0, 0%; PDX/GOS: 5, 6%; *P* = 0.027). Any medically confirmed adverse event was considered serious if it met one or more of the following criteria: resulted in death, was life-threatening, required inpatient hospitalization or prolongation of existing hospitalization, resulted in persistent or significant disability/incapacity, or was a congenital anomaly/birth defect. A total of three participants experienced serious adverse events (Control: *n* = 2, 2%; PDX/GOS: *n* = 1, 1%). All serious adverse events were individually evaluated by the study site physicians and each was determined to be unrelated to study formulas.

### Effects of diet on stool microbiome

**Fig. 4:**
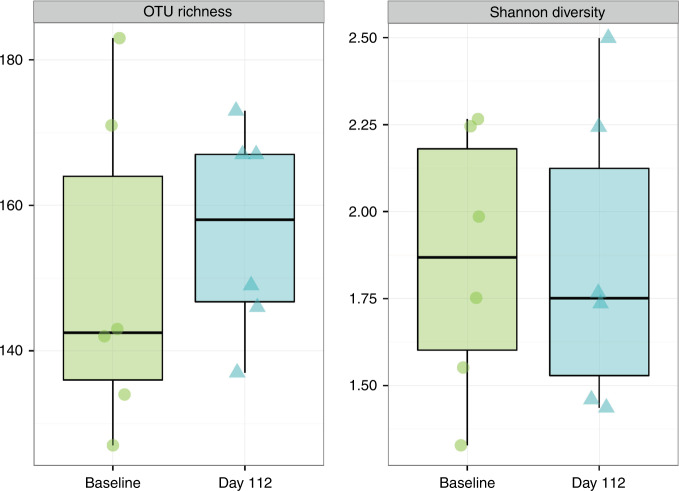
Alpha-diversity, PDX/GOS group. Exploratory stool microbiome alpha-diversity measures at Baseline and Day 112 in participants subset of the PDX/GOS group (*n* = 6).

**Fig. 5:**
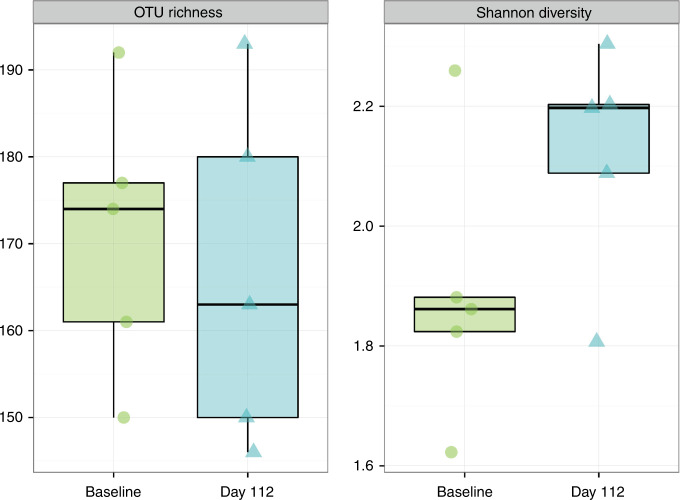
Alpha-diversity, Control group. Exploratory stool microbiome alpha-diversity measures at Baseline and Day 112 in participants subsets of the Control group (*n* = 5).

Stool samples at Baseline and Day 112 were analyzed in a subset of participants (Control, *n* = 5; PDX/GOS, *n* = 6). From Baseline to Day 112, no significant differences in OTU richness (sum of unique OTUs) or Shannon index (the number of OTUs in a sample relative to OTU abundance) were detected in either study group (Figs. 4, 5). No significant differences in beta diversity (Bray–Curtis distance, a measure of between-sample microbial community composition) from Baseline to Day 112 were detected by PERMANOVA (permutational multivariate analysis of variance) analysis for the Control group. However, a significant shift in beta diversity between Baseline and Day 112 was detected for the PDX/GOS group (PERMANOVA, *P* = 0.001). For the PDX/GOS group, the relative abundance of bacterial family *Lachnospiraceae* was significantly higher at Baseline compared to Day 112 (*P* = 0.036). For the Control vs. PDX/GOS group, by Day 112 the relative abundance of bacterial family *Coriobacteriaceae* was significantly higher (*P* = 0.02). A numerical increase in the genus *Bifidobacterium*, in which *Bifidobacterium* spp. became dominant by Day 112, was observed for the PDX/GOS group (Baseline vs. Day 112: mean percent relative abundance [standard deviation, SD] was 39.6 [20.7] and 57.7 [15.9], respectively). No statistically significant group differences were detected at the phylum level.

## Discussion

To the best of our knowledge, no previous studies have assessed the influence of prebiotics on development of human sleep–wake patterns. Results in the current study indicate faster consolidation of daytime waking state in infants receiving a prebiotic blend of PDX and GOS. Age-related behavioral maturation from Baseline to Day 112 was demonstrated for waking behavior. Shorter episodes of crying were also demonstrated by Day 70—a time point corresponding to the same age and developmental range typically associated with peak crying at ~2 months of age. The presence of CAR was demonstrated in all study participants.

Sleep is a time of intense brain activity in which onset and maintenance requires inhibition of the underpinning mechanisms controlling the waking state (and vice versa for onset and maintenance of the waking state). Given this reciprocal inhibition/activation process, sleep–wake patterns offer a window into the developing brain.^28^ Over the first year of life, the percentage of active sleep (the precursor of REM sleep) decreases and quiet sleep (non-REM sleep) increases. Results in the current study indicate faster consolidation of the sleep–wake cycle in infants receiving prebiotics. By Day 112, mean latency to the first and second diurnal nap onset was longer in the PDX/GOS group. Given that no group differences were detected for duration of first nap, results suggest earlier wake consolidation in the PDX/GOS group and more organized diurnal nap pattern. Differences in sleep–wake state changes with advancing age and between the diurnal and nocturnal periods, including both amount and time distribution, have been previously demonstrated.^29–31^

Infant temperament and psychomotor development has been inconsistently linked with sleep pattern development.^6,32,33^ This inconsistency may be due, in part, to the reliance upon sleep diaries rather than direct measures. Although accurate sleep–wake pattern data can be acquired through polysomnography (gold standard) in an academic sleep laboratory, recordings are expensive, very time-consuming, and modify the family’s usual routine. Infant sleep patterns can be evaluated through daily actigraph recordings, which are more precise than parent report^34,35^ and usually close with polysomnography conducted under clinical supervision in a sleep laboratory.^36^ Ambulatory actimetry instruments sensitive to movement, typically worn on the wrist or ankle that records activity over time (actigraphy), have been previously used in young infants for extended periods of time with good parental and infant compliance and accurate readings.^37,38^ The current study provides further support for use of home-based actigraphy to accurately assess sleep–wake patterns throughout the first months of life.

The presence of CAR and relationship with sleep has been evaluated previously in children at 12, 18, and 24 months of age.^39^ Although higher waking cortisol was associated with earlier awakening and shorter total sleep time, there were no significant differences in total sleep time, wake after sleep onset, number of awakenings, average length of awakening, efficiency, or awakening time between infants with or without the presence of CAR. By assessing nighttime along with daytime sleep–wake patterns, wake time and daytime napping have been demonstrated to induce modifications of CAR.^40,41^ Previous studies have suggested that emergence of CAR varies by individual and can appear from 2 to 6 months of age.^10,11^ Current study results confirm the presence of CAR by ~2 months of age.

In the present study, we assessed potential nutritive effects of specific dietary components (prebiotics) early in life on sleep–wake patterns in infants. Preliminary results, assessing stool samples in a subset of participants, have suggested that prebiotics in infant formula started early in life could support the healthy gut microbiome and positively affect sleep pattern development. Microbiome diversity is an amount of variation of microbial community within a sample (alpha diversity), or between samples (beta diversity). No significant group differences in alpha-diversity measures were demonstrated, suggesting that richness or evenness within individual participant samples were not impacted. In the PDX/GOS group, the numerical increase in the genus *Bifidobacterium* agrees with previous studies demonstrating PDX and GOS in infant formulas has a bifidogenic effect^17^ and the significant increase of family *Lachnospiraceae* may have potentially contributed to the observed beneficial effects, since this bacterial family includes butyrate-producing bacteria.^42^ Also, the family *Coriobacteriaceae* (demonstrated to increase significantly in the ceca of mice in response to stress) was not impacted in infants receiving the prebiotic blend, but was significantly increased in the Control group. Overall, the effect of prebiotics on wake organization is consistent with an influence on the gut–brain axis and requires further study with larger numbers of samples.

This study is limited by the lack of data regarding bedtime and nighttime parental practices, such as bedtime interactions, soothing routines, and socio-cultural context, to further define the role of these routines on the establishment of sleep–wake patterns and cortisol levels. In addition, some aspects of infant behavior study outcomes relied on parent-reported measures (Barr diary). Another limitation is measurement of cortisol, as an end-product of a neuroendocrine system, compared to measuring a precursor such as adrenocorticotropin, which could help understand potential mechanisms underlying CAR (e.g., hypothalamic or adrenal).

## Conclusion

Shorter episodes of crying were demonstrated by Day 70 in infants receiving formula with prebiotics, a time point corresponding to the same age and developmental range typically associated with peak crying. Results indicate faster consolidation of daytime waking state in infants receiving prebiotics and provide further support for home-based actigraphy to assess sleep–wake patterns throughout the first months of life. Results also suggest improved behavioral indicators of tolerance in infants receiving a prebiotic blend and further support the need to assess the connection of nutrition and infant state throughout the first months of life. The effect of prebiotics on infant state is consistent with an influence on the gut–brain axis and requires further study.

## Supplementary information

Supplementary figure
